# Fluid overload in the ICU: evaluation and management

**DOI:** 10.1186/s12882-016-0323-6

**Published:** 2016-08-02

**Authors:** Rolando Claure-Del Granado, Ravindra L. Mehta

**Affiliations:** 1 Hospital Obrero #2, Caja Nacional de Salud, Universidad Mayor de San Simón, School of Medicine, Avenida Blanco Galindo Km. 5 ½, Cochabamba, Bolivia; 2 School of Medicine, University of California San Diego, 200 West Arbor Drive #8342, San Diego, CA 92103 USA

**Keywords:** Fluid overload, Acute kidney injury, Diuretics, Continuous renal replacement therapies

## Abstract

**Background:**

Fluid overload is frequently found in acute kidney injury patients in critical care units. Recent studies have shown the relationship of fluid overload with adverse outcomes; hence, manage and optimization of fluid balance becomes a central component of the management of critically ill patients.

**Discussion:**

In critically ill patients, in order to restore cardiac output, systemic blood pressure and renal perfusion an adequate fluid resuscitation is essential. Achieving an appropriate level of volume management requires knowledge of the underlying pathophysiology, evaluation of volume status, and selection of appropriate solution for volume repletion, and maintenance and modulation of the tissue perfusion. Numerous recent studies have established a correlation between fluid overload and mortality in critically ill patients. Fluid overload recognition and assessment requires an accurate documentation of intakes and outputs; yet, there is a wide difference in how it is evaluated, reviewed and utilized. Accurate volume status evaluation is essential for appropriate therapy since errors of volume evaluation can result in either in lack of essential treatment or unnecessary fluid administration, and both scenarios are associated with increased mortality. There are several methods to evaluate fluid status; however, most of the tests currently used are fairly inaccurate. Diuretics, especially loop diuretics, remain a valid therapeutic alternative. Fluid overload refractory to medical therapy requires the application of extracorporeal therapies.

**Summary:**

In critically ill patients, fluid overload is related to increased mortality and also lead to several complications like pulmonary edema, cardiac failure, delayed wound healing, tissue breakdown, and impaired bowel function. Therefore, the evaluation of volume status is crucial in the early management of critically ill patients. Diuretics are frequently used as an initial therapy; however, due to their limited effectiveness the use of continuous renal replacement techniques are often required for fluid overload treatment. Successful fluid overload treatment depends on precise assessment of individual volume status, understanding the principles of fluid management with ultrafiltration, and clear treatment goals.

## Background

Fluid overload is frequently found in critically ill patients with acute kidney injury (AKI). Increasing fluid overload should not merely be considered an expected consequence of fluid resuscitation or severe AKI, it should be seen as a probably mediator of adverse outcomes. In critically ill patients, recent studies have highlighted the role of fluid overload on adverse outcomes [1]. Observational studies in pediatric patients who required continuous renal replacement therapy (CRRT) have shown an association between fluid overload and mortality [2–4]. Restrictive fluid management strategies are beneficial during acute respiratory distress syndrome and following major surgery since they reduce the duration of mechanical ventilation and the rate of cardiopulmonary complications [5, 6]. In concert with these data, the control and optimization of fluid balance is a key element of critically ill patients management, since inadequate fluid removal is associated with peripheral edema and pulmonary edema, which can retard weaning from mechanical ventilation, or compromise wound healing. We will focus on the evaluation and management of fluid overload in the intensive care unit (ICU).

## Discussion

### The role of fluid therapy in the development of fluid overload

In critically ill patients, adequate fluid resuscitation is essential to the restoration of cardiac output, systemic blood pressure and renal perfusion in patients with cardiogenic or septic shock [7, 8]. Prompt and adequate treatment with intravenous solutions can also prevent or limit subsequent AKI [9]. Achieving an appropriate level of volume management requires knowledge of the underlying pathophysiology, evaluation of volume status, selection of appropriate solution for volume repletion, and maintenance and modulation of the tissue perfusion [10].

The administration of crystalloids solutions that are recommend for the initial management of patients with or at risk of AKI, and also in patients with sepsis expands the extracellular compartment, but over time since critically ill patients have a increased capillary leak intravenous solutions will leave the circulation and distribute in the extracellular volume leading to edema and to fluid overload. These results in impaired oxygen and metabolite diffusion, distorted tissue architecture, obstruction of capillary blood flow and lymphatic drainage, and disturbed cell to cell interactions that may then contribute to progressive organ dysfunction (Table 1). These effects are prominent in encapsulated organs (liver and kidneys) [11–13]. Fluid overload is not only a consequence of fluid therapy but also occurs during severe sepsis secondary to the release of complement factors, cytokines and prostaglandin products and altered organ microcirculation [14]. In this context, edema is attributed to a combination of increased capillary permeability to proteins and increased net trans-capillary hydrostatic pressure through reduced pre-capillary vasoconstriction [15].

**Table 1:** Consequences of fluid overload in organ systems

Organ	Consequences
Cerebral edema	Impaired cognition
	Delirium
Myocardial edema	Conduction disturbance
	Impaired contractility
	Diastolic dysfunction
Pulmonary edema	Impaired gas exchange
	Reduced compliance
	Increased work of breathing
Renal interstitial edema	Reduced RBF
	Increased interstitial pressure
	Reduced GFR
	Uremia
	Salt and water retention
Hepatic congestion	Impaired synthetic function
	Cholestasis
Gut edema	Malabsorption
	Ileus
Tissue edema	Poor wound healing
	Wound infection
	Pressure ulceration

*RBF* renal blood flow, *GFR* glomerular filtration rate

### Fluid overload and outcomes

Several observational studies have demonstrated a correlation between fluid overload and mortality in critically ill patients with acute respiratory distress syndrome, acute lung injury, sepsis, and AKI. Bouchard et al., have shown that patients with fluid overload defined as an increase in body weight of over 10 % had significantly more respiratory failure, need of mechanical ventilation, and more sepsis. After adjusting for severity of illness, AKI patients with fluid overload had increased 30 day and 60 day mortality. Among survivors, AKI patients who required renal replacement therapy had a significantly lower level of fluid accumulation at initiation of dialysis and at dialysis cessation than non-survivors. Renal recovery was significantly lower in patients with fluid overload [1]. In children, a multicenter prospective study found that the percentage of fluid accumulation at initiation of CRRT was significantly lower in the survivors (14.2 % ±15.9 % vs. 25.4 % ±32.9 %, *P* = 0.03) [3].

Lungs are one of the organs in which adverse effects of fluid overload are most evident, which can lead to acute pulmonary edema or acute respiratory distress syndrome [16]. Several studies have provided evidence associating positive fluid balances with poorer respiratory outcomes. In one of these studies, septic shock patients with acute lung injury who received conservative fluid management after initial fluid resuscitation had lower in-hospital mortality [17]. In another study, Wiedemann et al. randomized 1000 patients to either a conservative or to a liberal strategy of fluid management. Patients randomized to the conservative fluid strategy had lower cumulative fluid balance, improved oxygenation index and lung injury score, increased number of ventilator-free days, and reduction in the length of ICU stay. It is worth to mention that the conservative fluid management strategy did not increase the incidence or prevalence of shock during the study or the need for renal replacement therapies [5]. Finally, in the Vasopressin in Septic Shock Trial (VASST) study authors found that higher positive fluid balance correlated significantly with increased mortality with the highest mortality rate observed in those with central venous pressure >12 mmHg [18].

### Fluid overload recognition and assessment

Fluid overload recognition and assessment in critically ill patients requires an accurate documentation of intakes and outputs; however, there is a wide variation in how this information is recorded, reviewed and utilized. Mehta RL and Bouchard J proposed some useful definitions to help us to standardize the approach and facilitated comparisons [10]:

1. ***Daily fluid balance***: daily difference in all intakes and all outputs, which frequently does not include insensible losses.
2. ***Cumulative fluid balance***: sum of each day fluid balance over a period of time.
3. ***Fluid overload***: usually implies a degree of pulmonary edema or peripheral edema.
4. ***Fluid accumulation***: positive fluid balance, with or without linked fluid overload.
5. ***Percentage of fluid overload adjusted for body weight***: cumulative fluid balance that is expressed as a percent. A cutoff of ≥10 % has been associated with increased mortality. Fluid overload percentage can be calculated using the following formula [19]:

$$ \%\ \mathbf{Fluid}\ \mathbf{overload}=\left(\left(\mathrm{total}\ \mathrm{fluid}\ \mathrm{in}-\mathrm{total}\ \mathrm{fluid}\ \mathrm{out}\right)/\mathrm{admission}\ \mathrm{body}\ \mathrm{weight}\times 100\right) $$

### Fluid status assessment

Accurate volume status evaluation is essential for appropriate therapy as inadequate assessment of volume status can result in not providing necessary treatment or in the administration of unneeded therapy, both associated with increased mortality. There are several methods to evaluate fluid status; however, most of the tests currently used are fairly inaccurate. We will describe some of these methods.

**Fig. 1:**
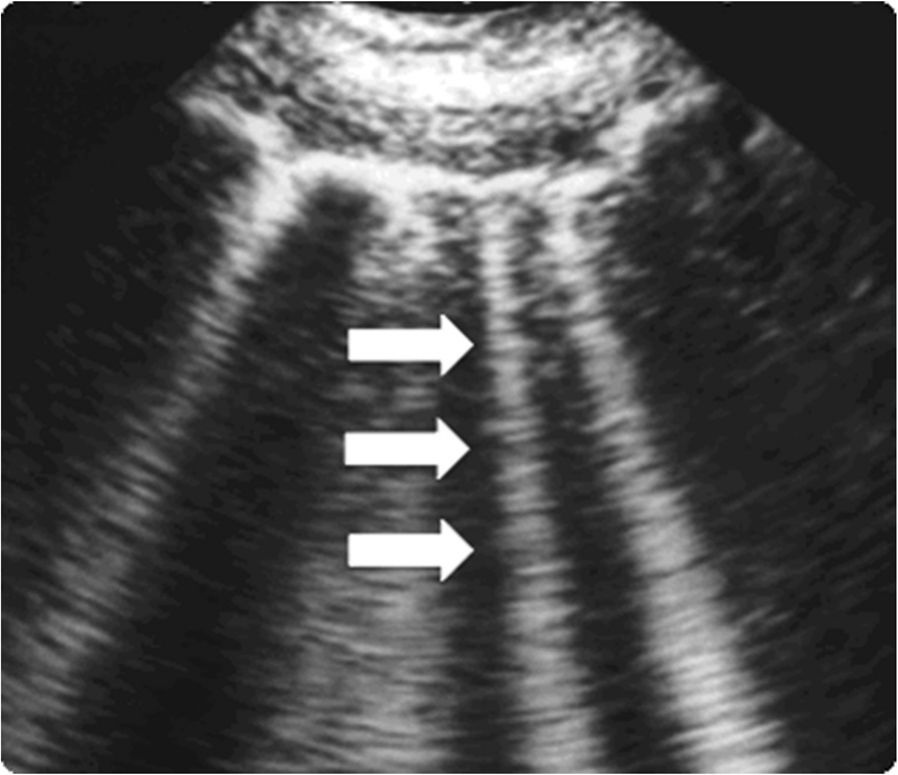
Lung comet tail image. ‘B lines’ also known as comet-tail images are a marker of pulmonary edema. In the presence of extravascular lung water the reflection of the ultrasound beam on the sub-pleural interlobular septa thickened by edema creates comet-tail reverberation artifacts. The ultrasound appearance is of a vertical, discrete, hyperechogenic image that arises from the pleural line and extends to the bottom of the screen moving synchronously with the respiration (white arrows)
- Vena cava diameter ultrasound
  The measurement of the inferior vena cava (IVC) diameter can also be use to assess volume status. Normal diameter of IVC is 1.5 to 2.5 cm (measured 3 cm from the right atrium); volume depletion is considered with an IVC diameter <1.5 cm while an IVC diameter >2.5 cm suggests volume overload.
  In an observational study on blood donors, Lyon et al. evaluated the inferior vena cava diameter (IVCd) during inspiration (IVCd*i*) and during expiration (IVCd*e*), before and after blood donation of 450 mL. Significant differences were found between the IVCd*e* before and after blood donation and between IVCd*i* before and after donation (5.5 mm and 5.16 mm, respectively) [34]. In patients treated for hypovolemia, Zengin et al. evaluated the IVC and right ventricle (RVd) diameters and diameter changes with the diameters and diameter changes of healthy volunteers. The IVCd was measured ultrasonographically by M-mode in the subxiphoid area and the RVd was measured in the third and fourth intercostals spaces before and after fluid resuscitation. As compare with healthy volunteers average diameters in hypovolemic patients of the IVC during inspiration and expiration, and right ventricule diameter were significantly lower. After fluid resuscitation, there was a significant increase in mean IVC diameters during inspiration and expiration as well as in the right ventricule diameter [35]. Bedside inferior vena cava diameter and right ventricule diameter evaluation could be a practical noninvasive instrument for fluid status estimation and for evaluating the response to fluid therapy in critically ill patients.

- History and physical examination:
  The usefulness of medical history, symptoms, and signs along with routine diagnostic studies (chest radiograph, electrocardiogram, and serum B-type natriuretic peptide (BNP)) that differentiate heart failure from other causes of dyspnea in the emergency department were evaluated in a meta-analysis. Many features increased the probability of heart failure, with the best feature for each category being the presence of past history of heart failure (positive LR = 5.8; 95 % CI, 4.1–8.0); paroxysmal nocturnal dyspnea (positive LR = 2.6; 95 % CI, 1.5–4.5); third heart sound gallop (positive LR = 11; 95 % CI, 4.9–25.0); chest radiograph showing pulmonary venous congestion (positive LR = 12.0; 95 % CI, 6.8–21.0); and electrocardiogram showing atrial fibrillation (positive LR = 3.8; 95 % CI, 1.7–8.8). A low serum BNP proved to be the most useful test (serum BNP <100 pg/mL; negative LR = 0.11; 95 % CI, 0.07–0.16) [20].
  Importantly, signs like pulmonary rales, lower extremity edema, and jugular venous distention have significant limits for assessing fluid overload. There are some studies that have correlated these sings during physical examination and invasive measures (e.g., pulmonary catheter wedge pressure (PCWP)). Butman et al. [21] found that the presence of jugular venous distension, at rest or inducible, had a sensitivity (81 %), and a specificity (80 %) for elevation of the pulmonary capillary wedge pressure (≥18 mmHg). Using hepato-jugular reflux and Valsalva maneuvers, Marantz et al. showed that these maneuvers were valid in the diagnosis of congestive heart failure in acutely dyspneic patients, with low a sensitivity (24 %) and a high specificity (94 %) [22].
  On the other hand, in a prospective study, physical signs of fluid overload were compared with hemodynamic measurements in 50 patients with known chronic heart failure. Sings like rales, edema, and elevated mean jugular venous pressures were absent in 18 of 43 patients with pulmonary capillary wedge pressures ≥22 mmHg. The combination of these signs had a sensitivity of 58 % and specificity of 100 % [23].
- Chest radiography
  Chest x-ray has been one of the most used tests to evaluate for hypervolemia. Radiographic sings of volume overload include dilated upper lobe vessels, cardiomegaly, interstitial edema, enlarged pulmonary artery, pleural effusion, alveolar edema, prominent superior vena cava, and Kerley lines. However, up to 20 % of patients diagnosed with heart failure had negative chest radiographs at initial emergency department evaluation. Additionally, these radiographic sings can be minimal in patients with late-stage heart failure [24].
  In patients with congestive heart failure, radiographic signs had poor predictive value for identifying patients with PCWP values ≥30 mmHg where radiographic pulmonary congestion was absent in 39 % of patients [25].
  The X-ray technique and the clinical status of patient impact radiographic performance for detecting volume overload. Portably chest X-ray, reduce the sensitivity of findings of volume overload [26], and pleural effusions can be missed if the film is performed supine. With intubated patients and patients with pleural effusions, the sensitivity, specificity, and accuracy of supine chest X-ray was reported to be as low as 60 %, 70 %, and 67 % respectively [27]. Conversely, the frequency of volume overload findings in the chest X-ray increased with the severity of fluid overload such as severe heart failure [28].
- Natriuretic peptides
  High levels of BNP can be found with volume overload; however, some conditions like myocardial infraction and pulmonary embolism can cause elevated levels of BNP. Other conditions that have to be taken into account when evaluating BNP levels are obesity, associated with lower BNP levels and renal failure, associated with high BNP levels. Patients with heart failure who have elevated base-line levels of BNP.
  The greatest utility of BNP levels is in the absence of elevation, since low BNP levels have a high negative predictive value for excluding heart failure diagnosis. On the other hand, high BNP levels can be non-specific for volume overload [26].
- Bioimpedance vector analysis
  Bioelectrical impedance analysis is a commonly used method for estimating body composition, specifically detecting soft tissue hydration with a 2–3 % measurement error. It is a noninvasive, inexpensive and highly versatile test that transforms electrical properties of tissues into clinical information [29]. Bioimpedance vector analysis (BIVA) measures whole body fluid volume and is based on patterns of the resistance-reactance graph, relating body impedance to body hydration [29]. Clinical information on hydration is obtained through patterns of vector distribution with respect to the healthy population of the same race, sex, class of body mass index, and age. Changes in tissue hydration status below 500 ml are detected and ranked. BIVA was examined as an indicator of fluid status compared to central venous pressure (CVP) in 121 critically ill patients [30]. In this study patients were classified in three groups according to their CVP value: low (0 to 3 mmHg); medium (4 to 12 mmHg); and high (13 to 20 mmHg). The agreement between BIVA and central venous pressure indications was good in the high CVP group, moderate in the medium CVP group, and poor in low CVP group. The combined evaluation of peripheral tissue hydration (BIVA) and central filling pressure (CVP) could provide a useful clinical assessment instrument in the planning of fluid therapy in critically ill patients, particularly in those with low CVP [31].
- Thoracic ultrasound
  Sonographic artifacts known as B-lines that suggest thickened interstitial or fluid-filled alveoli can be detected using thoracic ultrasound (Fig. 1). PCWP and fluid accumulation in lungs have been correlated with the presence of B-lines ("comet-tail images") in patients with congestive heart failure [32]. Agricola et al., used thoracic ultrasound to detect “comet-tail images” and obtained an individual patient comet-tail image score by summing the number of B-lines in each of the scanned spaces assessed (right and left hemi thorax, from second to fourth intercostals’ space, from para-sternal to mid-axillary line); authors found significant positive linear correlations between comet-tail images score and extra-vascular lung water determined by the PiCCO System, between comet score and PCWP, and between comet-tail images score and radiologic sings of fluid overload in the lungs [33].

### Fluid overload management

#### Diuretic therapy

Diuretics, especially loop diuretics, remain a valid therapeutic alternative for relieving symptoms and improving pathophysiological states of fluid overload such as congestive heart failure and in patients with AKI. At this time, there is no evidence that favors ultrafiltration over diuretic use in volume overload patients with or without AKI in terms of less progression of AKI, improved clinical outcomes or reduce incidence of AKI [36]. Despite that more patients developed AKI during diuretic treatment, numerous studies have demonstrated that more aggressive use of loop diuretics to achieve greater volume removal is associated with improved outcomes (Table 2) [37–40].

**Table 2:** Studies assessing the effect of diuretics on AKI and mortality

Study	Number	Comparison	Effect on AKI	Effect on mortality
Mehta et al. [37]	552	Diuretics versus no diuretics	Increased risk of death or non-recovery of renal function	OR 1.68 for death with diuretic use
Uchino et al. [38]	1743	Diuretics versus no diuretics	N/A	No difference
Cantarovich et al. [39]	338	Furosemide versus placebo	No difference on renal recovery were found	No difference
Grams et al. [40] FACTT trial	306	Fluid conservative (Furosemide dose of 80 mg) versus fluid liberal (Furosemide dose of 23 mg)	No difference in peak sCr	No difference

*FACTT* fluid and catheter treatment trial, *sCr* serum creatinine, *NA* non-assessed, *OR* odds ratio

What should be the goal of urine output when using diuretics to manage fluid overload? Some empirical observations have shown that a urine output of 3–4 ml/kg/h rarely causes intravascular volume depletion as capillary refill can meet such rates in almost all patients [41]. Diuretics could be either administered by bolus or using a continuous infusion. There has been controversy about which of these strategies is better; some authors advocate that diuretic infusion is superior to boluses since urinary output could be maintain easily [41]. In one study diuretic infusion was associated with greater diuresis and this was achieved with a lesser dose [42]; infusion was also associated with fewer adverse events such as worsening AKI, hypokalemia, and ototoxicity. However, in the DOSE-AHF(Diuretic Optimization Strategy Evaluation in Acute Decompensated Heart Failure) study, authors found that patients with acute heart failure may benefit from an initial bolus strategy [43].

Since common electrolyte disturbances could be encountered during diuretic therapy, it is important to monitor electrolytes levels and also to assess acid-based status. In order to avoid hypokalemia, administration of oral potassium it is easy. Measuring urinary potassium concentration and calculating the daily losses of potassium, which require replacement is a strategy that can be used to estimate daily potassium requirements. Another strategy is the use of potassium-sparing diuretics like spironolactone. Hypomagnesemia is frequently found during diuretic therapy, magnesium replacement can be achieved either intravenously or orally, typically with 20–30 mmoL per day. Finally in some patients, chloride losses exceed sodium losses and hypochloremic metabolic alkalosis develops; this is usually corrected with the administration of potassium chloride and magnesium chloride.

A recent comprehensive review have shown that torsemide and bumetanide have more favorable pharmacokinetic profiles than furosemide, and in the case of torsemide it could be more efficacious than furosemide in patients with heart failure (decreased mortality, decrease hospitalizations, and improved New York Heart Association functional classification). In AKI patients, as compared with torsemide the use of furosemide was associated with a significant improvement in urine output. Moreover, two trials comparing bumetanide with furosemide showed conflicting results [44].

Finally, in patients with AKI the response to furosemide may be reduced due to multiple mechanisms including a reduced tubular secretion of furosemide and blunted response of Na-K-2Cl co-transporters at the loop of Henle [45]. This reduced response to furosemide in AKI patients often requires the use of higher doses that may increase the risk of ototoxicity, especially as the clearance of furosemide is severely reduced in AKI. High doses of furosemide may also result in myocardial dysfunction secondary to furosemide induced vasoconstriction [46].

#### Extracorporeal therapies

Fluid overload refractory to medical therapy requires the use of extracorporeal therapies such as continuous renal replacement therapies since critically ill patients often show hemodynamic instability and/or multiple organ dysfunctions. Accurate management of fluid balance becomes obligatory with the ultimate goal of improving pulmonary gas exchange and organ perfusion while maintaining stable hemodynamic parameters. The optimal renal replacement therapy for patients with AKI and fluid overload has not been defined yet and there is still an ongoing debate. Choice of the initial modality needs to be based on the availability of resources, local expertise; the individual needs of the patients, and finally on patient’s hemodynamic status.

In patients with fluid overload, CRRT provides a slower fluid removal over intermittent hemodialysis (IHD) resulting in more hemodynamic stability and better fluid balance control, other advantages of CRRT over IHD include: a slower control of solute concentration avoiding large fluctuations and fluid shifts, which reduce the risk of cerebral edema, the great flexibility in terms of treatment adjustment to patient’s needs at anytime, and finally CRRT allows to perform the treatment with relatively simple and user friendly machines [47]. Some large observational studies have suggested that CRRT is an independent predictor of renal recovery among survivors [48–50].

In the absence of definite data to support the use of particular type of renal replacement therapy, one should consider CRRT and IHD as complementary therapies. Therefore, during the treatment of critically ill patients with AKI and fluid overload transitions between CRRT and IHD are frequent, and are frequently driven by patients’ hemodynamic status.

Slow continuous ultrafiltration (SCUF) is a type of continuous renal replacement therapy that is usually performed with low blood flow rates (50 to 100 ml/min), and ultrafiltration rates between 100 and 300 ml/h according to fluid balance necessities. Relatively small surface-area filters can be employed with reduced heparin doses since low ultrafiltration and blood flow rates are required, [51].

Continuous veno-venous hemofiltration (CVVH) is another CRRT technique that allows meticulous, minute-to-minute control of fluid balance by providing continuous fluid, electrolyte, and toxin clearance.

**Fig. 2:**
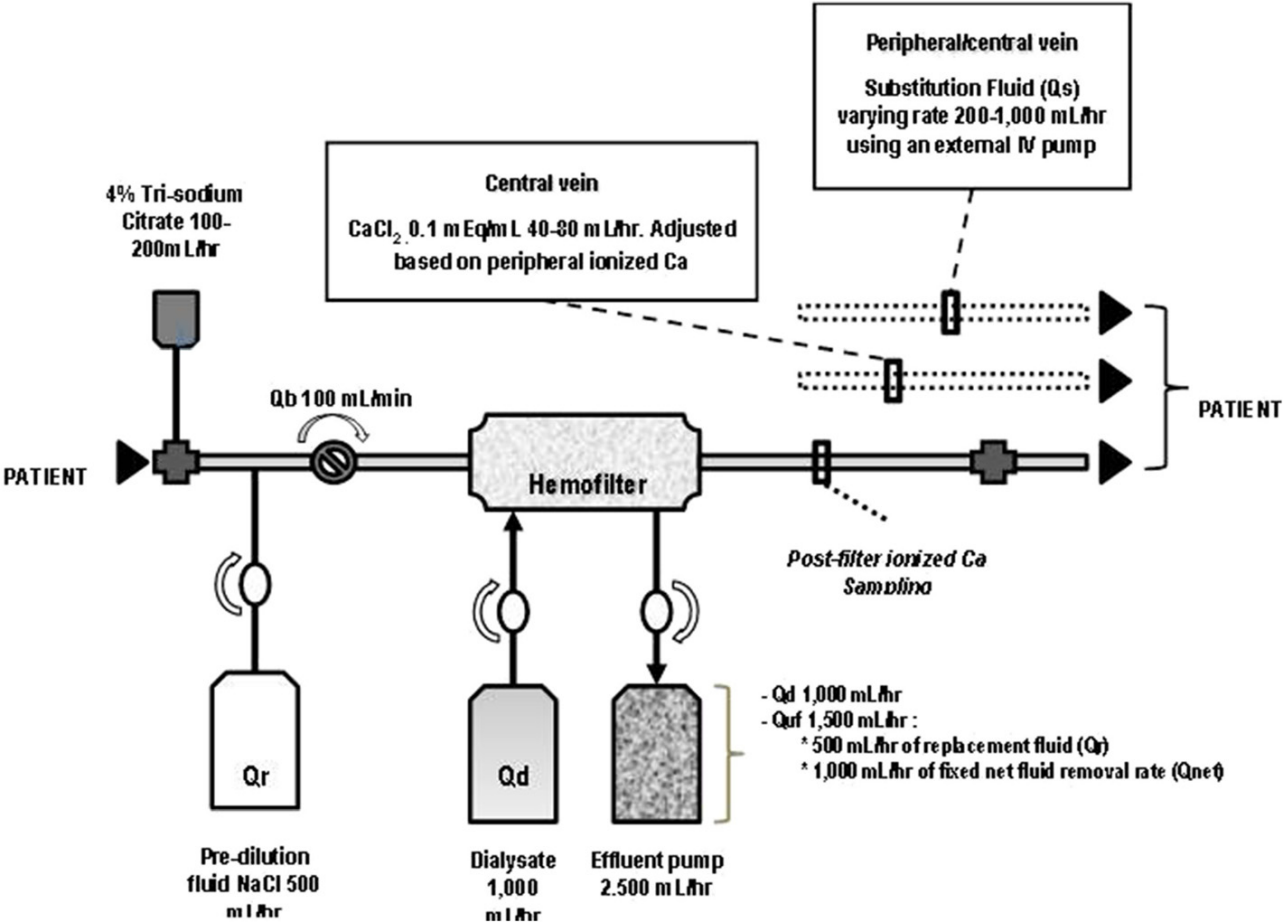
Circuit set up at University of California San Diego, Medical Center. The mean infusion rate of tri-sodium citrate was 180 ml/h and blood flow rate (Qb) was set at 100 ml/min. Tri-sodium citrate was added at the arterial catheter port with ionized calcium levels been measured post-filter. Post-filter ionized calcium levels were used to adjust tri-sodium citrate flow rates. Pre-filter BUN value was measured after the infusion of tri-sodium citrate and after pre-dilution replacement fluid (Qr), thus accounting for the pre-dilutional effect. A fixed ultrafiltration rate (Quf) was used (set at 1000 ml/h) for achieving fluid balance. A target effluent volume was adjusted by hourly modifying substitution fluid rate (Qs) to achieve a negative, zero, or positive fluid balance. Qb, blood flow rate; Qd, dialysate flow rate; Qr, replacement fluid rate; Quf, total ultrafiltration rate; Qnet, net fluid removal rate

The prescription of CRRT related fluid management and its integration into overall patient fluid management could be improved by using a specific order chart for the machine fluid balance as shown on Table 3. Machine fluid balance refers to the total balance over 24-h period of fluids administered by the CRRT machine (dialysate or replacement fluid or both depending on the technique) and fluids removed by the CRRT machine (spent dialysate or ultrafiltrate or both depending on the technique). This set up will help to achieve the planned hourly fluid balance as shown on Table 3 and Fig. 2.

**Table 3:** Order chart for achieving hourly fluid balance

Technique	Dialysate flow rate (Qd)	Replacement fluid flow rate (Qr)	Ultrafiltration flow rate (Quf)	Effluent flow rate (Qeff)	Substitution fluid flow rate (Qs) *using an external IV pump*	Machine fluid balance (NetUF)
^a^CVVHDF	1000 mL/h	500 mL/h	1000 mL/h	2500 mL/h	Varying rate from 200 to 1000 mL/h,	−300 mL/h

^a^ *CVVHDF* continuous veno-venous hemodiafiltration

The ultimate goal is to preserve tissue perfusion, optimizing fluid balance by effectively removing fluid without compromising the effective circulating fluid volume; therefore, meticulous monitoring of fluid balance is critical for all patients [52].

Another option for treating patients with fluid overload are the new smaller and more portable devices like the Aquadex FlexFlow System (Baxter Healthcare). In patients with heart failure, Costanzo et al. compare adjustable ultrafiltration using a small ultrafiltration device to the use of intravenous loop diuretics. The authors found a trend to longer time to recurrence of heart failure within 90 days event after hospital discharge in patients treated with the ultrafiltration device, and fewer heart failure and cardiovascular events. Changes in renal function and the 90-day mortality were similar in both groups. However, more patients who were randomized to adjustable ultrafiltration experienced an adverse effect of special interest (*p* = 0.018) and a serious study product-related adverse events (*p* = 0.026) [53].

## Conclusions

Several complications like congestive heart failure, pulmonary edema, delayed wound healing, tissue breakdown, and impaired bowel function are associated with fluid overload. Fluid overload has also been related to increased mortality. The optimal assessment of volume status in critically ill patients is of vital importance particularly during the early management of these patients. One key aspect of fluid overload management is to maintain hemodynamic stability and optimize organ function. Loop diuretics are frequently used as the initial therapy to treat critically ill patients with fluid overload; nevertheless, diuretics have limited effectiveness due to several factors such as underlying acute kidney injury that contribute to diuretic resistance. Renal replacement therapies are often required for optimal volume management in critically ill patients with fluid overload. In this setting, successful volume management depends on an accurate estimation of patients’ fluid status, an adequate understanding of the principles of fluid overload treatment with ultrafiltration, and clear treatment goals.

## Abbreviations

AKI, acute kidney injury; BIVA, Bio-impedance vector analysis; BNP, B-type natriuretic peptide; CRRT, continuous renal replacement therapy; CVP, central venous pressure; CVVH, continuous veno-venous hemofiltration; EVLW, extra-vascular lung water; ICU, intensive care unit; IHD, intermittent hemodialysis; IVC, inferior vena cava; IVCd, inferior vena cava diameter; IVCde, inferior vena cava diameter during expiration; IVCdi, inferior vena cava diameter during inspiration; PCWP, pulmonary catheter wedge pressure; RVd, right ventricle diameter; SCUF, slow continuous ultrafiltration

## References

[1] Bouchard J, Soroko SB, Chertow GM, Himmelfarb J, Ikizler TA, Paganini EP (2009). Fluid accumulation, survival and recovery of kidney function in critically ill patients with acute kidney injury. Kidney Int.

[2] Goldstein SL, Currier H, Graf C, Cosio CC, Brewer ED, Sachdeva R (2001). Outcome in children receiving continuous venovenous hemofiltration. Pediatrics.

[3] Goldstein SL, Somers MJ, Baum MA, Symons JM, Brophy PD, Blowey D (2005). Pediatric patients with multi-organ dysfunction syndrome receiving continuous renal replacement therapy. Kidney Int.

[4] Gillespie RS, Seidel K, Symons JM (2004). Effect of fluid overload and dose of replacement fluid on survival in hemofiltration. Pediatr Nephrol.

[5] Wiedemann HP, Wheeler AP, Bernard GR, Thompson BT, Hayden D, deBoisblanc B (2006). Comparison of two fluid-management strategies in acute lung injury. N Engl J Med.

[6] Brandstrup B, Tonnesen H, Beier-Holgersen R, Hjortso E, Ording H, Lindorff-Larsen K (2003). Effects of intravenous fluid restriction on postoperative complications: comparison of two perioperative fluid regimens: a randomized assessor-blinded multicenter trial. Ann Surg.

[7] Prowle JR, Echeverri JE, Ligabo EV, Ronco C, Bellomo R (2010). Fluid balance and acute kidney injury. Nat Rev Nephrol.

[8] Levy MM, Artigas A, Phillips GS, Rhodes A, Beale R, Osborn T (2012). Outcomes of the Surviving Sepsis Campaign in intensive care units in the USA and Europe: a prospective cohort study. Lancet Infect Dis.

[9] Kellum JA, Lameire N. Kidney Disease Improving Global Outcomes (KDIGO) Working Group. Section 3: Prevention and Treatment of AKI. Kidney Int Suppl (2011). 2012;2(1):37–68. 10.1038/kisup.2011.33 PMC4089747 25018919

[10] Mehta RL, Bouchard J (2011). Controversies in acute kidney injury: effects of fluid overload on outcome. Contrib Nephrol.

[11] Humphrey H, Hall J, Sznajder I, Silverstein M, Wood L (1990). Improved survival in ARDS patients associated with a reduction in pulmonary capillary wedge pressure. Chest.

[12] Nisanevich V, Felsenstein I, Almogy G, Weissman C, Einav S, Matot I (2005). Effect of intraoperative fluid management on outcome after intraabdominal surgery. Anesthesiology.

[13] Boyle A, Maurer MS, Sobotka PA (2007). Myocellular and interstitial edema and circulating volume expansion as a cause of morbidity and mortality in heart failure. J Card Fail.

[14] Andreucci M, Federico S, Andreucci VE (2001). Edema and acute renal failure. Semin Nephrol.

[15] Bouchard J, Mehta RL (2010). Fluid balance issues in the critically ill patient. Contrib Nephrol.

[16] Schrier RW, Wang W (2004). Acute renal failure and sepsis. N Engl J Med.

[17] Murphy CV, Schramm GE, Doherty JA, Reichley RM, Gajic O, Afessa B (2009). The importance of fluid management in acute lung injury secondary to septic shock. Chest.

[18] Boyd JH, Forbes J, Nakada TA, Walley KR, Russell JA (2011). Fluid resuscitation in septic shock: a positive fluid balance and elevated central venous pressure are associated with increased mortality. Crit Care Med.

[19] Bagshaw SM, Cruz DN (2010). Fluid overload as a biomarker of heart failure and acute kidney injury. Contrib Nephrol.

[20] Wang CS, FitzGerald JM, Schulzer M, Mak E, Ayas NT (2005). Does this dyspneic patient in the emergency department have congestive heart failure?. JAMA.

[21] Butman SM, Ewy GA, Standen JR, Kern KB, Hahn E (1993). Bedside cardiovascular examination in patients with severe chronic heart failure: importance of rest or inducible jugular venous distension. J Am Coll Cardiol.

[22] Marantz PR, Kaplan MC, Alderman MH (1990). Clinical diagnosis of congestive heart failure in patients with acute dyspnea. Chest.

[23] Stevenson LW, Perloff JK (1989). The limited reliability of physical signs for estimating hemodynamics in chronic heart failure. JAMA.

[24] Collins SP, Lindsell CJ, Storrow AB, Abraham WT (2006). Prevalence of negative chest radiography results in the emergency department patient with decompensated heart failure. Ann Emerg Med.

[25] Chakko S, Woska D, Martinez H, de Marchena E, Futterman L, Kessler KM (1991). Clinical, radiographic, and hemodynamic correlations in chronic congestive heart failure: conflicting results may lead to inappropriate care. Am J Med.

[26] Peacock WF, Soto KM (2010). Current techniques of fluid status assessment. Contrib Nephrol.

[27] Ruskin JA, Gurney JW, Thorsen MK, Goodman LR (1987). Detection of pleural effusions on supine chest radiographs. AJR Am J Roentgenol.

[28] Chait A, Cohen HE, Meltzer LE, VanDurme JP (1972). The bedside chest radiograph in the evaluation of incipient heart failure. Radiology.

[29] Piccoli A (2002). Patterns of bioelectrical impedance vector analysis: learning from electrocardiography and forgetting electric circuit models. Nutrition.

[30] Piccoli A, Pittoni G, Facco E, Favaro E, Pillon L (2000). Relationship between central venous pressure and bioimpedance vector analysis in critically ill patients. Crit Care Med.

[31] Piccoli A (2010). Bioelectric impedance measurement for fluid status assessment. Contrib Nephrol.

[32] Picano E, Frassi F, Agricola E, Gligorova S, Gargani L, Mottola G (2006). Ultrasound lung comets: a clinically useful sign of extravascular lung water. J Am Soc Echocardiogr.

[33] Agricola E, Bove T, Oppizzi M, Marino G, Zangrillo A, Margonato A (2005). “Ultrasound comet-tail images”: a marker of pulmonary edema: a comparative study with wedge pressure and extravascular lung water. Chest.

[34] Lyon M, Blaivas M, Brannam L (2005). Sonographic measurement of the inferior vena cava as a marker of blood loss. Am J Emerg Med.

[35] Zengin S, Al B, Genc S, Yildirim C, Ercan S, Dogan M (2013). Role of inferior vena cava and right ventricular diameter in assessment of volume status: a comparative study: ultrasound and hypovolemia. Am J Emerg Med.

[36] Perazella MA, Coca SG (2013). Three feasible strategies to minimize kidney injury in ‘incipient AKI’. Nat Rev Nephrol.

[37] Mehta RL, Pascual MT, Soroko S, Chertow GM (2002). Diuretics, mortality, and nonrecovery of renal function in acute renal failure. JAMA.

[38] Uchino S, Doig GS, Bellomo R, Morimatsu H, Morgera S, Schetz M (2004). Diuretics and mortality in acute renal failure. Crit Care Med.

[39] Cantarovich F, Rangoonwala B, Lorenz H, Verho M, Esnault VL (2004). High-dose furosemide for established ARF: a prospective, randomized, double-blind, placebo-controlled, multicenter trial. Am J Kidney Dis.

[40] Grams ME, Estrella MM, Coresh J, Brower RG, Liu KD (2011). Fluid balance, diuretic use, and mortality in acute kidney injury. Clin J Am Soc Nephrol.

[41] Bellomo R, Prowle JR, Echeverri JE (2010). Diuretic therapy in fluid-overloaded and heart failure patients. Contrib Nephrol.

[42] Martin SJ, Danziger LH (1994). Continuous infusion of loop diuretics in the critically ill: a review of the literature. Crit Care Med.

[43] Shah RV, McNulty S, O’Connor CM, Felker GM, Braunwald E, Givertz MM (2012). Effect of admission oral diuretic dose on response to continuous versus bolus intravenous diuretics in acute heart failure: an analysis from diuretic optimization strategies in acute heart failure. Am Heart J.

[44] Wargo KA, Banta WM (2009). A comprehensive review of the loop diuretics: should furosemide be first line?. Ann Pharmacother.

[45] Brater DC (1981). Resistance to diuretics: emphasis on a pharmacological perspective. Drugs.

[46] De Vecchis R, Ciccarelli A, Cioppa C (2012). Intermittent intravenous infusion of high-dose loop diuretics and risk for iatrogenic ototoxicity: an unresolved issue from the DOSE study. G Ital Cardiol (Rome).

[47] Kellum JA, Lameire N. Kidney Disease Improving Global Outcomes (KDIGO) Working Group.Section 5: Dialysis Interventions for Treatment of AKI. Kidney Int Suppl (2011). 2012;2(1):89–115. 10.1038/kisup.2011.35 PMC4089702 25018921

[48] Bell M, Granath F, Schon S, Ekbom A, Martling CR, SWING (2007). Continuous renal replacement therapy is associated with less chronic renal failure than intermittent haemodialysis after acute renal failure. Intensive Care Med.

[49] Jacka MJ, Ivancinova X, Gibney RT (2005). Continuous renal replacement therapy improves renal recovery from acute renal failure. Can J Anaesth.

[50] Uchino S, Bellomo R, Kellum JA, Morimatsu H, Morgera S, Schetz MR (2007). Patient and kidney survival by dialysis modality in critically ill patients with acute kidney injury. Int J Artif Organs.

[51] Cerda J, Ronco C (2009). Modalities of continuous renal replacement therapy: technical and clinical considerations. Semin Dial.

[52] Bouchard J, Mehta RL (2009). Volume management in continuous renal replacement therapy. Semin Dial.

[53] Costanzo MR, Negoianu D, Jaski BE, Bart BA, Heywood JT, Anand IS (2016). Aquapheresis Versus Intravenous Diuretics and Hospitalizations for Heart Failure. JACC Heart Fail.

